# Differences in corneal phenotypes between destrin mutants are due to allelic difference and modified by genetic background

**Published:** 2012-03-03

**Authors:** Sharolyn V. Kawakami-Schulz, Angela M. Verdoni, Shannon G. Sattler, Akihiro Ikeda, Sakae Ikeda

**Affiliations:** Department of Medical Genetics, University of Wisconsin, Madison, WI

## Abstract

**Purpose:**

Mutations in destrin (*Dstn*) cause corneal abnormalities in mice. A null mutation, *Dstn^corn1^*, results in corneal epithelial hyperproliferation, inflammation, and neovascularization in the A.BY background (A.BY *Dstn^corn1^*). Homozygosity for a point mutation, *Dstn^corn1–2J^*, results in mild thickening of the corneal epithelium but no corneal neovascularization in a C57BL/6 (B6) background (B6 *Dstn^corn1–2J^*). The goal of this study was to determine whether phenotypic differences are due to allelic differences between *Dstn^corn1^* and *Dstn^corn1–2J^*, or are the result of genetic background effects.

**Methods:**

We generated two congenic (Cg) mouse lines, B6.Cg-*Dstn^corn1^* and A.BY.Cg-*Dstn^corn1–2J^*, to compare to the original A.BY *Dstn^corn1^* and B6 *Dstn^corn1–2J^* lines. We performed immunohistochemistry to assay F-actin accumulation, neovascularization, proliferation, and inflammation. By western blot analysis we tested the expression of serum response factor (SRF), a known regulator of the *Dstn^corn1^* phenotype.

**Results:**

The *Dstn^corn1^* mutation leads to neovascularization, hyperproliferation, and inflammation in the cornea of A.BY *Dstn^corn1^* as well as B6.Cg-*Dstn^corn1^* mice. We did not observe significant corneal neovascularization or hyperproliferation in either A.BY.Cg-*Dstn^corn1–2J^* or B6 *Dstn^corn1–2J^* mice. Actin accumulation, neovascularization, epithelial proliferation and inflammation in B6.Cg-*Dstn^corn1^* cornea are significantly reduced when compared to A.BY *Dstn^corn1^*cornea. SRF changes are consistent in *Dstn^corn1^* mutants, regardless of genetic background.

**Conclusions:**

Differences in the abnormal phenotypes of *Dstn* mutants result from allelic differences between *Dstn^corn1^* and *Dstn^corn1–2J^* . Moreover, phenotypes of *Dstn^corn1^* mice are modified by genetic background, suggesting the existence of genetic modifiers. Protein analysis suggests that a genetic modifier affects phenotypic severity functionally downstream from or in a pathway independent from SRF. These data demonstrate that natural genetic variation affects phenotypic severity in *Dstn^corn1^* mice.

## Introduction

Mutations in the gene for the actin depolymerizing factor destrin (DSTN), lead to abnormalities in the structure and function of the cornea and subsequent blindness in mice [1-3]. Since actin depolymerizing factors including DSTN function to maintain a homeostatic ratio of actin in two forms, filamentous actin (F-actin) and globular actin (G-actin) [4], *Dstn* mutant mice demonstrate the importance of actin dynamics regulation to the proper structure and functions of the cornea. We have previously reported both phenotypic and gene expression differences between two mutations of the *Dstn* gene, *Dstn^corn1^* and *Dstn^corn1–2J^* [2,3]. The *Dstn^corn1^* mutation is characterized by deletion of a ~35 Kb region containing the entire coding sequence for *Dstn*, while the *Dstn^corn1–2J^* allele has been identified as a point mutation in exon 3, resulting in a Proline to Serine amino acid change in the actin binding domain [3]. *Dstn^corn1^* was isolated and has been maintained in the A.BY genetic background, while *Dstn^corn1–2J^* has been propagated in the C57BL/6 (B6) background.

There are four major classes of phenotypes that distinguish the two *Dstn* mutations. First, while both mutants display abnormal levels of F-actin accumulation in the corneal epithelium, consistent with the role of DSTN to depolymerize F-actin, A.BY *Dstn^corn1^* mice display extreme misregulation of actin dynamics, leading to structural breakdown of the corneal epithelium. B6 *Dstn^corn1–2J^* mice display mild F-actin accumulation but maintain overall structural integrity of the corneal epithelium [3]. Second, while A.BY *Dstn^corn1^* mice develop neovascularization into the cornea, B6 *Dstn^corn1–2J^* cornea remain free of any abnormal vasculature. Third, the A.BY *Dstn^corn1^* mutants demonstrate increased epithelial thickening due to proliferating cells throughout the corneal epithelium when compared to B6 *Dstn^corn1–2J^* [1,3]. Fourth, A.BY *Dstn^corn1^* mutants displayed significant recruitment of inflammatory cells to the corneal epithelium when compared to B6 *Dstn^corn1–2J^* [5]. Since these mutations are on different genetic backgrounds, it was impossible to deduce whether phenotypic differences between *Dstn^corn1^* and *Dstn^corn1–2J^* were solely due to differences in mutant alleles, or if the mutants are differentially affected because of genetic backgrounds.

To observe each mutation independently of background effects, we used the original lines, A.BY *Dstn^corn1^* and B6 *Dstn^corn1–2J^*, to create a pair of congenic (Cg) lines, B6.Cg-*Dstn^corn1^* and A.BY.Cg-*Dstn^corn1–2J^* . By comparing A.BY *Dstn^corn1^* to A.BY.Cg-*Dstn^corn1–2J^* and B6 *Dstn^corn1–2J^* to B6.Cg-*Dstn^corn1^*, we are able to assess the allelic differences between the two mutants independent of genetic background effects. Moreover, by comparing A.BY *Dstn^corn1^* to B6.Cg-*Dstn^corn1^* and A.BY.Cg-*Dstn^corn1-2J^* to B6 *Dstn^corn1-2J^*, we can observe the effects of genetic background on the disease phenotypes attributed to different types of mutations. These analyses demonstrated that both allelic difference and genetic background affect the severity of corneal abnormalities caused by the *Dstn* mutation.

## Methods

### Mouse husbandry

A.BY-*H2^b^* H2-*T18^b^*/SnJ (A.BY WT), C57BL/6J WT (B6 WT), A. BY-*H2^b^* H2*T18^b^*/SnJ-*Dstn^corn1^*/J (A.BY *Dstn^corn1^*), and C57BL/6JSmn-*Dstn^corn1–2J^* (B6 *Dstn^corn1–2J^*) mice were obtained from the Jackson Laboratory (Bar Harbor, ME) and bred in an animal facility at the University of Wisconsin-Madison. All mouse procedures were performed in accordance with the protocols approved by the Animal Care and Use Committee at the University of Wisconsin-Madison and conform to the Association for Research in Vision and Ophthalmology (ARVO) statement for the use of animals in ophthalmic and vision research.

### Generation of congenic mouse lines

B6.Cg-*Dstn^corn1^* mice were generated by crossing A.BY *Dstn^corn1^* mice to B6 WT mice, selecting for mice carrying a *Dstn^corn1^* allele, and backcrossing to B6 WT for 8 generations. Heterozygous mice were intercrossed and progeny genotyped as homozygous for the *Dstn^corn1^* mutation were used for analysis. A.BY.Cg-*Dstn^corn1-^*^2J^ mice were generated by crossing B6 *Dstn^corn1–2J^* mice to A.BY WT mice, selecting for mice carrying a *Dstn^corn1–2J^* allele, and backcrossing to A.BY WT for 10 generations. Heterozygous mice were intercrossed and progeny genotyped as homozygous for the *Dstn^corn1–2J^* mutation were used for analysis.

### Genotyping

All mice were genotyped using polymerase chain reaction (PCR). To genotype for *Dstn^+^*, primers mADF-F31 (5′-GTC CCA TGA ATG TGA ATT GC-3′) and mADF-R28 (5′-CCC TGG TGA CCT TTC CTT ATC-3′) were used for amplification. To genotype for *Dstn^corn1^*, primers mADF-F32 (5′-GCC ACA TCA TTA GCT TTT GAA G3′) and mADF-R30 (5′-TGG CAC TCC TGC TGT CAC-3′) were used for amplification. To genotype for *Dstn^corn1–2J^*, primers mADF-F35 (5′TGG AGG GTG TGC TTT CTC TAC-3′) and mADF-R32 (5′-CTA CGA AGA TAA TAA GGT GGG C-3′) were used for amplification, followed by digestion of the PCR product with the restriction enzyme BanI.

### Immunohistochemistry

All mice were analyzed at postnatal day 58 (P58), unless otherwise indicated. Immunohistochemistry on frozen sections was performed as described previously [2]. Briefly, after removal, eyes were fixed in paraformaldehyde and gradients of sucrose before sectioning. Sections were blocked with phosphate-buffered saline (PBS) with 0.5% Triton X and 2% normal donkey serum before overnight incubation with primary antibodies. Sections were then rinsed with PBS, incubated with a secondary antibody, and stained with DAPI. The primary antibodies and dilutions used for the analysis were Ki67 (1:100; Thermo Scientific, Fremont, CA), myeloperoxidase (1:200; R&D Systems, Minneapolis, MN), and cluster of differentiation 45 (CD45; 1:100; BD Pharmigen, San Diego, CA). Sections were incubated with Alexa Fluor 488 or 568 conjugated secondary antibody (1:400; Invitrogen, Carlsbad, CA) or Alexa Fluor 568 conjugated phalloidin (1:50; Invitrogen) and were counterstained with 4,6-diamidino-2-phenylindole dihydrochloride (DAPI, 1:200; Sigma-Aldrich, St. Louis, MO).

Immunohistochemistry on whole cornea was performed as described previously [2]. Briefly, after removal of the eye, corneas were removed and fixed in paraformaldehyde overnight. Corneas were then rinsed in PBS and post-fixed in 100% acetone, followed by additional PBS rinses. Corneas were blocked overnight in PBS with 0.8% Triton and 2% normal donkey serum, followed with an overnight incubation with the primary antibody in block solution. The primary antibody for cluster of differentiation 31 (CD31; BD Pharmigen) was used at a dilution of 1:50. Corneas were transferred to block solution containing phalloidin (1:50; Invitrogen) and an Alexa Fluor 488 conjugated secondary antibody (1:200; Invitrogen). Corneas were counterstained with DAPI (1:200; Sigma-Aldrich).

### Imaging

Images acquired on sections and whole mount cornea were captured on an Eclipse E600 microscope (Nikon, Tokyo, Japan) using a SPOT camera (Spot Diagnostics, Sterling Heights, MI) or a Zeiss 510 confocal laser scanning system and Axio Imager microscope using LSM 510 software (release 4.2; Carl Zeiss MicroImaging, Thornwood, NY). For images obtained of the surface of eye, mice were sacrificed and immediately placed under the objective of a Zeiss Stemi SV11 dissecting scope attached to a Nikon COOLPIX 995 digital camera (Nikon).

### Western blotting

For each line, six corneas pooled from three mice were homogenized in RIPA buffer (1× PBS with 1% NP-40 and 0.1%SDS) containing a protease inhibitor cocktail. Protein concentrations were determined using the Precision Red protein assay reagent (Cytoskeleton, Denver, CO) according to the manufacturer’s instructions. Equal amount of protein were subjected to SDS–PAGE using 4%–12% Bis-tris gels and antibodies against SRF (1:1,000; Santa Cruz Biotenchnology, Santa Cruz, CA), and FMR1 (1:4,000; Millipore, Billerica, MA). Blots were treated with horseradish peroxidase conjugated secondary antibodies (1:2,000; Jackson Immunoresearch, West Grove, PA) before detection using a chemiluminescent reagent (Amersham ECL Plus western blotting detection system; General Electric, Buckinghamshire, UK). Blots were then exposed to X-ray film (Thermo Scientific, Rockford, IL). Western blot analyses were performed in both biologic and technical triplicate. FMR1 (fragile X mental retardation syndrome 1 homolog) was used as a loading control, as previously described [6].

### Analysis of *Dstn^corn1^*/*Dstn^+^* relative density ratio

The *Dstn^corn1^* relative density/*Dstn^+^* relative density measurements were performed via scanning densitometry using ImageJ software. Bands identified as SRF were normalized against their respective FMR1 control bands. Normalized values, obtained in biologic triplicate, were then pooled for analysis.

### Histological quantification of proliferating cells and inflammatory cells

Corneal frozen sections were stained for the proliferation marker Ki67, the pan-leukocyte marker CD45, the neutrophil marker myeloperoxidase (MP), and the nuclear marker DAPI. Cells were counted using ImageJ software on digital images taken using the SPOT Image Analysis system. Two separate, nonconsecutive sections were analyzed for each eye for each phenotype.

### Quantification of the vascularized area of the cornea

Digital images of all corneal flat mounts were collected using the Spot Image Analysis system. Vascularized area and total corneal area were measured using ImageJ software with a method similar to that of Bock et al. [7]. Briefly, filters were applied to subtract background, reduce noise, and enhance contrast. The total corneal area was outlined using the innermost vessel of the limbal arcade as the border, and the area of CD31-positive vessels within the cornea was then calculated and normalized to the total corneal area (expressed as the percentage of vascularized cornea) using thresh holding analysis. Filters were applied to subtract background, reduce noise, and enhance contrast. The total corneal area was outlined using the innermost vessel of the limbal arcade as the border, and the area of CD31-positive vessels within the cornea was then calculated and normalized to the total corneal area (expressed as the percentage of vascularized cornea) using threshholding analysis.

### Statistical analysis

For comparison of three groups, a one-way ANOVA was performed, followed by pairwise *t*-tests with a Bonferroni correction if the ANOVA resulted in a p<0.05. For comparison of two groups, a two-tailed, unpaired *t-*test was used. GraphPad Prism software (GraphPad, San Diego, CA) was used for statistical analysis and to create all graphs reporting numerical values. *p<0.05, **p<0.01, ***p<0.001.

## Results

Characterization of genetic mutations in the gene for DSTN and phenotypic differences in the cornea of A.BY *Dstn^corn1^* and B6 *Dstn^corn1–2J^* had been reported previously [1,3,5]. In this study, we created the congenic lines B6.Cg-*Dstn^corn1^* and A.BY.Cg-*Dstn^corn1–2J^* to compare to the original lines. *Dstn^corn1^* mice lack the coding region for DSTN and represent a null mutant of the *Dstn* protein, while *Dstn^corn1–2J^* mice are characterized by a non-synonymous mutation in the actin binding domain of DSTN that most likely results in hypomorphic activity.

**Figure 1:**
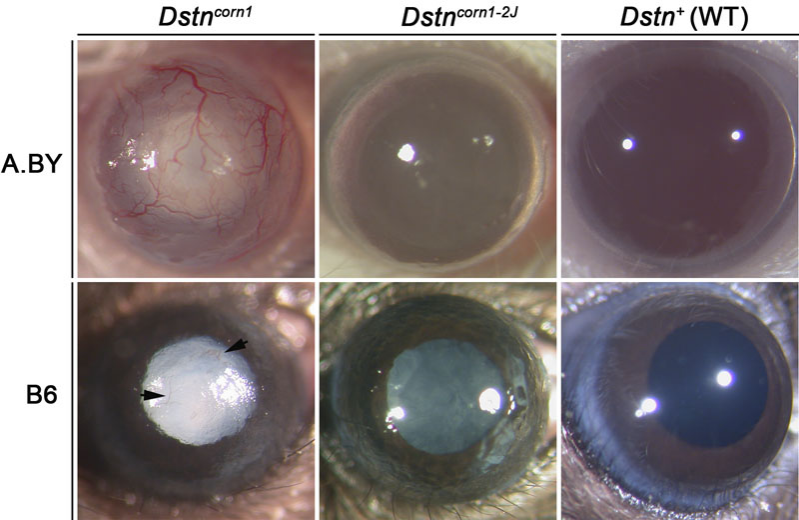
Gross anatomic imaging of eyes of A.BY *Dstn^corn1^*, B6.Cg-*Dstn^corn1^* , A.BY.Cg-*Dstn^corn1–2J^*, B6 *Dstn^corn1–2J^*, A.BY WT, and B6 WT mice. Corneal neovascularization occurs only in lines with the *Dstn^corn1^*mutation (arrowheads in B6.Cg-*Dstn^corn1^*). While all *Dstn* mutants display a roughened corneal surface, this phenotype is more severe in *Dstn^corn1^*. Cataract formation occurs in all four mutants, but is delayed in *Dstn^corn1–2J^* when compared to *Dstn^corn1^*.

Even upon gross visual analysis, these two *Dstn* mutations exhibit phenotypic differences at P58 (Figure 1). The corneal surface of A.BY *Dstn^corn1^* and B6.Cg-*Dstn^corn1^* mice is roughened and blood vessels are clearly visible. Cataracts in the lens are another characteristic feature of *Dstn^corn1^* eyes in both backgrounds at P58. The corneal surface of A.BY.Cg-*Dstn^corn1–2J^* and B6 *Dstn^corn1–2J^* also appears rough when compared to WT, but to a lesser degree. In mice with the *Dstn^corn1–2J^* mutation, a cataract has not fully formed by P58 but develops in all mice as they age (data not shown). Additionally, neither *Dstn^corn1-^*^2J^ mutant displays corneal neovascularization (Figure 1).

**Figure 2:**
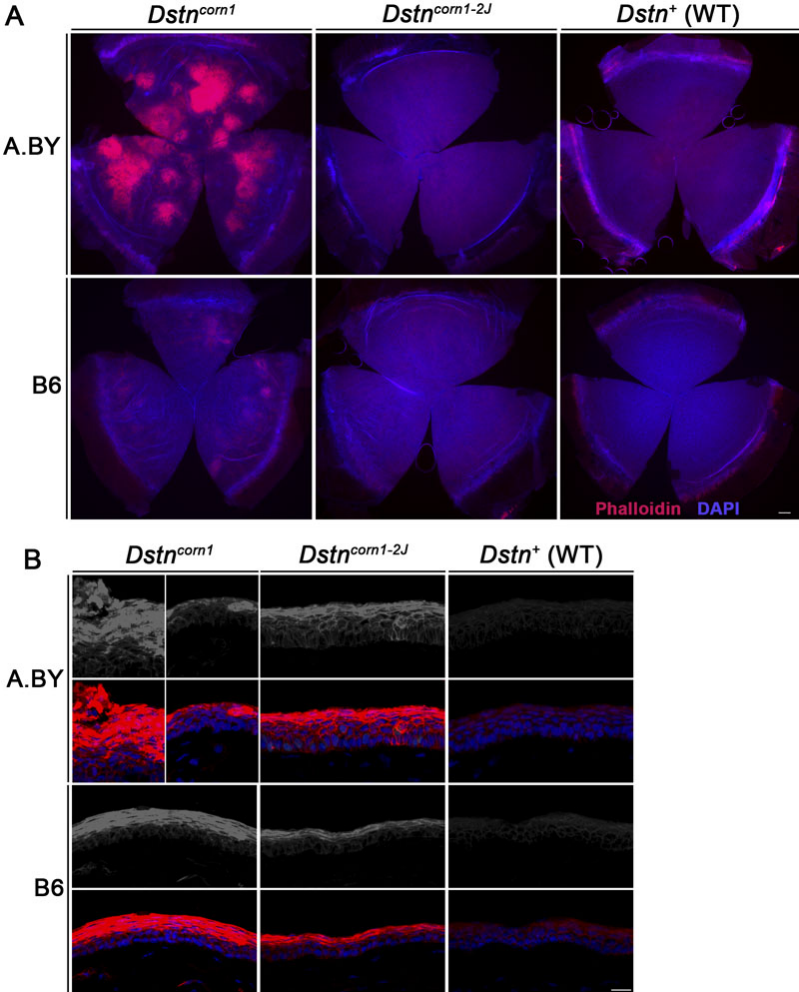
F-actin in the cornea of *Dstn* mutants and WT mice in both the A.BY and B6 background at P58. **A**: Phalloidin staining of whole mount cornea at P58 shows that F-actin (red) accumulation more severe in *Dstn^corn1^* cornea compared to *Dstn^corn1–2J^* cornea in both the A.BY and B6 background. F-actin accumulation is less severe in the cornea of B6.Cg-*Dstn^corn1^* when compared to A.BY *Dstn^corn1^* . Bar, 200 μm. **B**: Phalloidin staining of frozen sections illustrates the degree of F-actin (gray in upper panels, red in lower panels) accumulation in each *Dstn* mutant compared to WT. Two portions of an A.BY *Dstn^corn1^* cornea are shown to represent its irregular surface. Bar, 20 μm. All slides were counterstained with DAPI to mark cell nuclei (blue).

By staining the whole mount cornea and frozen sections of the cornea with phalloidin, which specifically binds to F-actin, we compared F-actin accumulation among the four lines of mice to WT. We observed that, even when comparing the mutations in the same background (A.BY *Dstn^corn1^*vs A.BY.Cg-*Dstn^corn1–2J^* and B6.Cg-*Dstn^corn1^* versus B6 *Dstn^corn1–2J^*), *Dstn^corn1–2J^* displays milder F-actin accumulation compared to *Dstn^corn1^* . The level of F-actin accumulation in the B6.Cg-*Dstn^corn1^* cornea is less severe compared to the A.BY *Dstn^corn1^*cornea (Figure 2).

**Figure 3:**
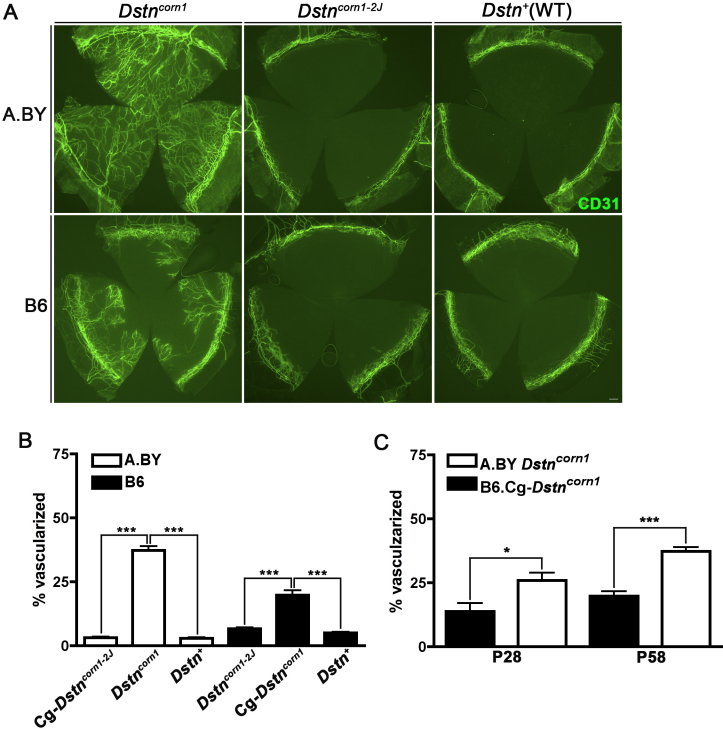
Corneal neovascularization caused by the *Dstn^corn1^* mutation. **A**: Immunofluorescence for CD31 highlights blood vessels that infiltrate *Dstn^corn1^* cornea in both the A.BY and B6 background. Significant neovascularization is not observed as a result of the *Dstn^corn1–2J^* mutation, making the appearance similar to WT. Bar, 200 μm. **B**: Quantification of the vascularized area shows that *Dstn^corn1^* cornea have significantly more vasculature compared to *Dstn^corn1–2J^* and WT cornea in both genetic backgrounds (p<0.001 for both backgrounds). Note that the resting level of vasculature is higher in B6 than A.BY. **C**: Genetic background effect on the neovascularization phenotype in the *Dstn^corn1^* cornea is significant at postnatal day 28, and becomes even more significant with age. Sample sizes: A.BY.Cg-*Dstn^corn1–2J^* P58 n=5, A.BY *Dstn^corn1^* P58 n=11, A.BY WT P58 n=3, B6 *Dstn^corn1–2J^* P58 n=4, B6.Cg-*Dstn^corn1^* P58 n=10, B6 WT P58 n=4, A.BY *Dstn^corn1^* P28 n=11, B6.Cg-*Dstn^corn1^* P28 n=6. Error bars, SEM * denotes statistical significance resulting from *t*-tests, with omitted bars representing nonsignficance. *p<0.05, **p<0.01, ***p<0.001.

To examine the amount of corneal neovascularization, we performed immunofluorescence using an anti-CD31 antibody on whole cornea. Quantification of the vascularized area in A.BY *Dstn^corn1^* to A.BY.Cg-*Dstn^corn1–2J^* and B6.Cg-*Dstn^corn1^* to B6 *Dstn^corn1–2J^* and WT mice revealed that significant corneal neovascularization occurs only in mice with the *Dstn^corn1^* mutation, in both the A.BY and B6 backgrounds. There is no significant difference in the amount of vasculature in *Dstn^corn1–2J^* compared to WT (Figure 3A,B). Neovascularization is significantly reduced in the cornea of B6.Cg-*Dstn^corn1^* mice compared to A.BY *Dstn^corn1^* mice. While this difference is apparent and significant by P28, the difference increases in significance with age (Figure 3C).

**Figure 4:**
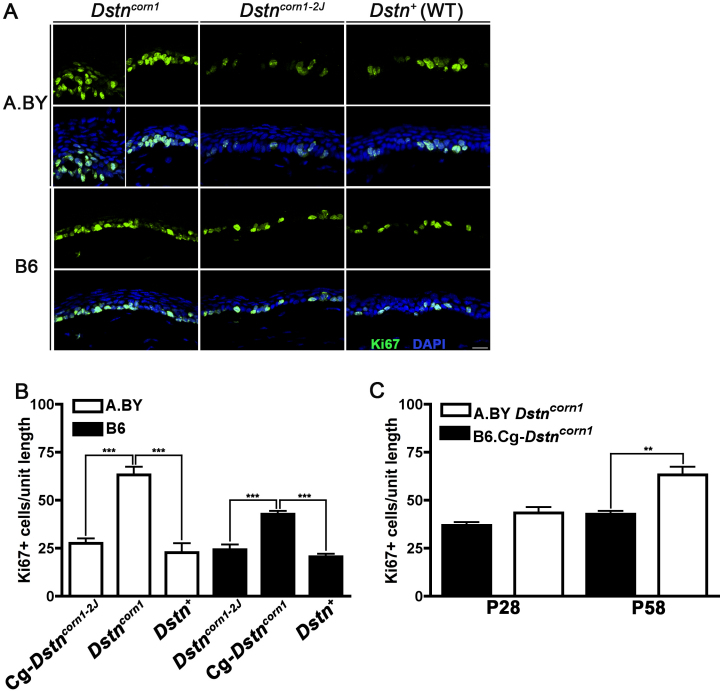
Epithelial hyperproliferation in *Dstn^corn1^* corneas. **A**: Immunofluorescence for Ki67 (green) labels proliferating cells in the corneal epithelium of *Dstn* mutants and WT mice on A.BY and B6 backgrounds at P58. All slides were counterstained with DAPI to mark cell nuclei (blue), which is shown merged with Ki67 staining in the lower panels. Bar, 20 μm. **B**: Quantification of Ki67 positive cells shows significantly increased numbers of proliferating cells in *Dstn^corn1^* cornea compared to *Dstn^corn1–2J^* and WT cornea in both A.BY and B6 backgrounds at P58 (p<0.001 for both backgrounds). **C**: A tendency for increased hyperproliferation in A.BY *Dstn^corn1^* compared to B6.Cg-*Dstn^corn1^* is observed by P28. This difference becomes statistically significant by P58. Sample sizes: A.BY.Cg-*Dstn^corn1–2J^* P58 n=4, A.BY *Dstn^corn1^* P58 n=5, A.BY WT P58 n=4, B6 *Dstn^corn1–2J^* P58 n=3, B6.Cg-*Dstn^corn1^* P58 n=5, B6 WT P58 n=4, A.BY *Dstn^corn1^* P28 n=6, B6.Cg-*Dstn^corn1^* P28 n=3. Unit length=300 μm. Error bars, SEM * denotes statistical significance resulting from *t*-tests, with omitted bars representing non-significance. *p<0.05, **p<0.01, ***p<0.001.

To detect proliferating cells in the corneal epithelium, we performed immunohistochemistry using an anti-Ki67 antibody. We compared the amount of proliferation in each of the four lines and found that the number of proliferating cells in the corneal epithelia of *Dstn^corn1^* mice is significantly more than that in the corneal epithelia of *Dstn^corn1–2J^* mice in both the A.BY and B6 backgrounds (Figure 4A,B). Hyperproliferation was significantly increased in the corneal epithelia of A.BY *Dstn^corn1^* mice compared to B6.Cg-*Dstn^corn1^* mice (Figure 4C). The trend of increased hyperproliferation in the corneal epithelium of A.BY *Dstn^corn1^* compared to B6.Cg-*Dstn^corn1^* is present by P28 and becomes significant by P58 (Figure 4C). In contrast, when comparing these phenotypes between A.BY.Cg-*Dstn^corn1–2J^* and B6 *Dstn^corn1–2J^* mice, we did not observe any significant difference.

**Figure 5:**
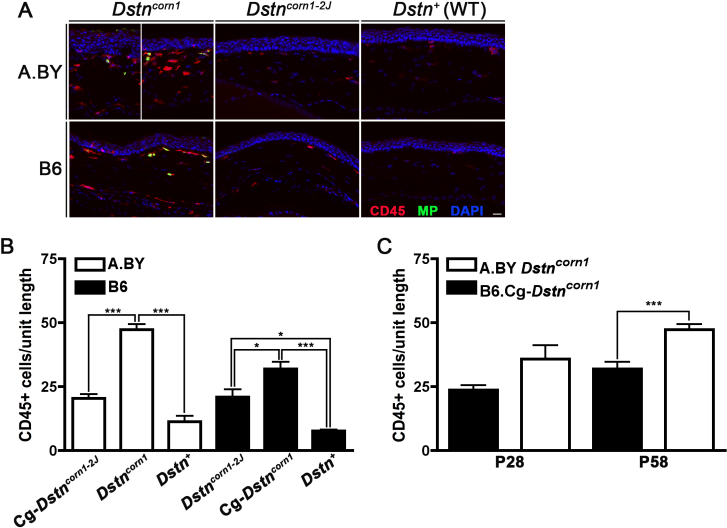
Recruitment of inflammatory cells to the cornea of *Dstn* mutants. **A**: Immunofluorescence using CD45 (red) to mark inflammatory cells and myeloperoxidase to mark neutrophils specifically in *Dstn* mutant and WT cornea on A.BY and B6 backgrounds at P58. All slides were counterstained with DAPI to mark cell nuclei (blue). Bar, 20 μm. **B**: Quantification of CD45 positive cells revealed significantly increased inflammation in both *Dstn^corn1^* and *Dstn^corn1–2J^* mutant cornea compared to WT at P58 (p<0.001 for both backgrounds). **C**: The trend for increased inflammation in A.BY *Dstn^corn1^* compared to B6.Cg-*Dstn^corn1^* is present by P28 and increases to significance by P58. Sample sizes: A.BY.Cg-*Dstn^corn1-2J^* P58 n=4, A.BY *Dstn^corn1^* P58 n=8, A.BY WT P58 n=4, B6 *Dstn^corn1–2J^* P58 n=4, B6.Cg-*Dstn^corn1^* P58 n=6, B6 WT P58 n=4, A.BY *Dstn^corn1^* P28 n=6, B6.Cg-*Dstn^corn1^* P28 n=3. Unit length=300 um. Error bars, SEM * denotes statistical significance resulting from *t*-tests, with omitted bars representing non-significance. *p<0.05, **p<0.01, ***p<0.001.

To detect inflammatory cells in the corneal epithelium, we performed immunohistochemistry using anti-CD45 and anti-myeloperoxidase antibodies. We compared the amount of inflammation in the cornea of mice from each of the four lines. We found that there is significantly increased recruitment of inflammatory cells to the cornea of *Dstn^corn1^* mice when compared to *Dstn^corn1–2J^* or WT mice, an observation common to both backgrounds. There is also a significant increase in inflammatory cells in *Dstn^corn1–2J^* mutant corneas compared to WT (Figure 5A,B). The level of inflammation was significantly increased in the cornea of A.BY *Dstn^corn1^* mice compared to B6.Cg-*Dstn^corn1^* mice (Figure 5C). In the corneal epithelium of *Dstn^corn1^* mice, the trend of increased inflammation in the A.BY compared to the B6 background is seen at P28 and becomes significant by P58 (Figure 5C). In contrast, when comparing these phenotypes between A.BY.Cg-*Dstn^corn1–2J^* and B6 *Dstn^corn1–2J^* mice, we did not observe any significant difference.

**Figure 6:**
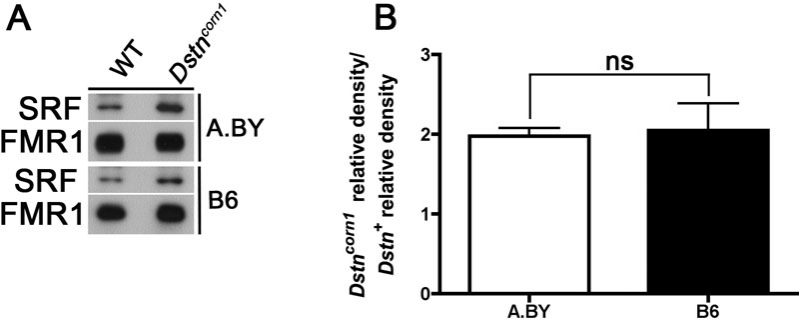
Western blot analysis for SRF in *Dstn^corn1^* mutant and WT cornea. **A**: SRF level is greater in *Dstn^corn1^* mutant cornea compared to WT. **B**: When comparing the induction of SRF expression by the *Dstn^corn1^* mutation (SRF increase in *Dstn^corn1^* with respect to WT) in both the A.BY and B6 background, there is no significant difference in the increase of SRF between these genetic backgrounds. Error bars, SEM * denotes statistical significance resulting from *t*-tests. *p<0.05, **p<0.01, ***p<0.001, ns=nonsignificant.

We had previously identified the transcription factor serum response factor (SRF) as a major contributor to *Dstn^corn1^* phenotypes [2,6]. To assess the quantity of SRF in the *Dstn^corn1^* mutants in both genetic backgrounds, we performed western blot analysis. We found that the level of SRF is higher in *Dstn^corn1^* when compared WT in both backgrounds (Figure 6A). Additionally, statistical analysis reveals that the level of induction of SRF expression in *Dstn^corn1^* mutants, that is, the increase relative to WT, is not significantly different between genetic backgrounds. (Figure 6B).

**Figure 7:**
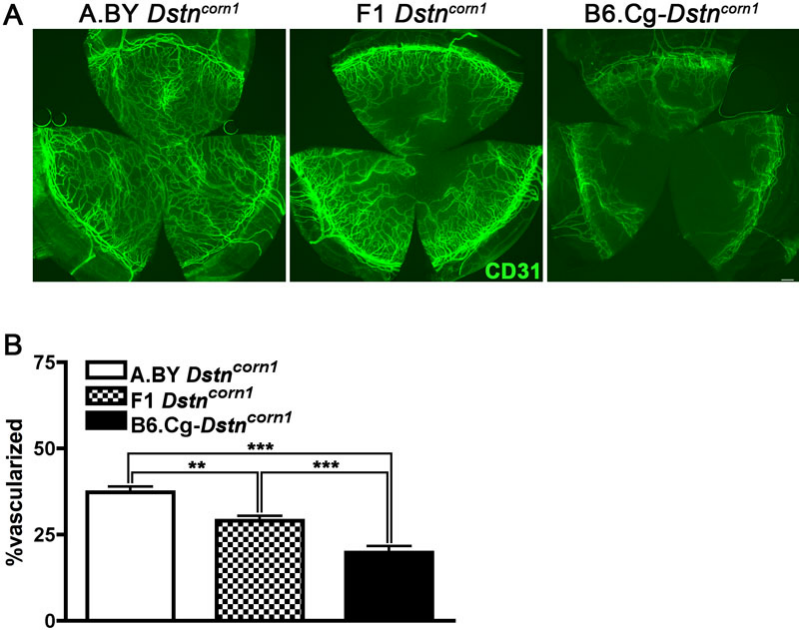
Neovascularization phenotype in F1 *Dstn^corn1^* progeny. **A**: Immunofluorescence for CD31 highlights blood vessels that infiltrate A.BY *Dstn^corn1^*, B6.Cg-*Dstn^corn1^*, and F1 (A.BY *Dstn^corn1^* x B6.Cg-*Dstn^corn1^*) cornea. Bar, 200 μm. **B**: The amount of neovascularization in F1 *Dstn^corn1^* mice is intermediate to and significantly different from either parental strain. Sample sizes: A.BY *Dstn^corn1^* P58 n=11, F1 *Dstn^corn1^* P58 n=14, B6.Cg-*Dstn^corn1^* n=10. Error bars, SEM * denotes statistical significance resulting from *t*-tests. *p<0.05, **p<0.01, ***p<0.001.

Our results suggest that phenotypic differences between *Dstn* mutants are due to allelic differences. Actin accumulation, neovascularization, proliferation and inflammatory response differences between *Dstn^corn1^* and *Dstn^corn1–2J^* remain consistent when compared in two genetic backgrounds. Notably, phenotypes caused by the *Dstn^corn1^*mutation are also modified by genetic background. In every case, the abnormal phenotype is dampened in the cornea of B6.Cg-*Dstn^corn1^* mice compared to A.BY *Dstn^corn1^* mice. We crossed the *Dstn^corn1^* mutants and found that F1 mice demonstrate a level of neovascularization that is intermediate to and significantly different from A.BY *Dstn^corn1^* and B6.Cg-*Dstn^corn1^* (Figure 7A,B). These results suggest the presence of a genetic factor that is variable between B6 and A.BY and affects the phenotype in a dosage-dependent manner.

## Discussion

### *Dstn^corn1^* and *Dstn^corn1–2J^* contribute to observed phenotypic differences

By observing corneal phenotypes on the same genetic background, this study shows that the differences in abnormal phenotypes that we observe in *Dstn* mutant mice are due to the allelic difference between *Dstn^corn1^* and *Dstn^corn1–2J^* . These observations are consistent with our previous finding that *Dstn^corn1^* is a null mutation, while *Dstn^corn1–2J^* is a point mutation [3]. Since the point mutation in *Dstn^corn1–2J^* leads to an amino acid change in the actin binding region of DSTN, the protein resulting from the *Dstn^corn1–2J^* mutation likely has residual functions leading to the milder phenotypes in *Dstn^corn1–2J^* cornea compared to *Dstn^corn1^* cornea where no DSTN protein is produced.

### Genetic modification is specific to the *Dstn^corn1^* mutation

The cornea of B6.Cg-*Dstn^corn1^* mice display milder forms of each phenotype when compared to A.BY *Dstn^corn1^* . This modification is not seen when comparing the cornea of B6 *Dstn^corn1–2J^* to A.BY.Cg-*Dstn^corn1–2J^*, suggesting the existence of genetic modifiers that specifically interact with molecules or pathways affected by the *Dstn^corn1^* mutation. In fact, our previous microarray study to compare A.BY *Dstn^corn1^* and B6 *Dstn^corn1–2J^* to their WT counterparts revealed that the *Dstn^corn1^* mutation led to differential expression of 1,226 annotated genes, whereas the *Dstn^corn1–2J^* mutation led to changes in only 202 annotated genes [2]. Based on these observations, we speculate that the modifier or modifiers may belong to or interact with the set of ~1000 genes whose differential expression is observed in *Dstn^corn1^* and not in *Dstn^corn1–2J^* . Another possible reason which could explain genetic modification specific to the *Dstn^corn1^* mutation is the involvement of a compensating mechanism that may be activated by a complete loss of destrin (*Dstn^corn1^*) but not by a point mutation (*Dstn^corn1–2J^*). There are 2 other members of the actin depolymerizing factor family in mice, cofilin 1 which is ubiquitously expressed and cofilin 2 which is muscle specific [8]. We previously observed upregulation of cofilins in the A.BY *Dstn^corn1^*cornea, indicating the existence of the compensating mechanism by cofilins [3]. It is possible that compensation by cofilins is more effective in the B6 background compared to the A.BY background, leading to milder phenotypes in the B6.Cg-*Dstn^corn1^* cornea compared to the A.BY *Dstn^corn1^*cornea.

### Effects of genetic variation on the mouse cornea

Genetic background effects have been observed for various phenotypes in the mouse cornea. The rate of wound healing, along with epithelial migration, was found to be greater in the cornea of B6 mice compared to BALB/c mice [9]. Induced corneal neovascularization [10,11] as well as lymphangiogenesis [12,13] has also been shown to be greatly affected by the genetic background. Central corneal thickness, which is known to be associated with the risk for glaucoma, differs among inbred strains of mice [14] and quantitative trait locus (QTL) analysis identified a genetic locus that is significantly associated with this phenotype [15]. Our data demonstrates that genetic background has a significant effect on the severity of *Dstn^corn1^* phenotypes, indicating the presence of a QTL that interacts with this mutation. We subsequently found that the F1 neovascularization phenotype is intermediate to both *Dstn^corn1^* mutants, suggesting that the locus or loci may act in a semi-dominant manner. Future identification of genetic factors responsible for these phenotypic differences in the cornea of *Dstn^corn1^* mice should provide valuable information regarding the molecular networks that regulate homeostasis and pathogenesis in the cornea.

### The genetic modifier of *Dstn^corn1^* acts in a pathway independent of SRF

Notably, all known phenotypes caused by the *Dstn^corn1^* mutation are similarly affected by genetic background. This observation suggests that genetic factors responsible for these phenotypic differences act on the part of a molecular pathway that affects all of these phenotypes. We previously identified one transcription factor that appears to be responsible for all phenotypes caused by the *Dstn^corn1^* mutation. Gene expression analysis revealed that multiple molecules that are downstream of the transcription factor SRF, as well as SRF itself, are abnormally upregulated in the cornea of *Dstn^corn1^* mice [2]. Inactivation of SRF specifically in the corneal epithelium of the *Dstn^corn1^* mice rescued all phenotypes caused by this mutation [6]. Data from this study suggest that the level of induction of SRF in *Dstn^corn1^* mutants does not appear to be directly responsible for the variation in severity of the phenotypes. That is, since the levels of induction of SRF in A.BY *Dstn^corn1^* and B6.Cg-*Dstn^corn1^* are not significantly different, we conclude that there exists an additional regulator responsible for the dampening of phenotypes in the B6 background. Since genetic factors responsible for phenotypic differences between A.BY *Dstn^corn1^* and B6.Cg-*Dstn^corn1^* mice affect all phenotypes in a similar manner, we hypothesize that these genetic factors may act downstream from SRF in a molecular pathway, or else function in an independent manner. Further investigation of the SRF pathway along with identification of these genetic factors will test this possibility.

### Conclusions

This study has shown that phenotypic differences between the *Dstn* mutants *Dstn^corn1^* and *Dstn^corn1–2J^* are due to allelic differences. Additionally, further analysis revealed that phenotypes caused by the *Dstn^corn1^* mutation are modified by genetic background. Our work indicates that there are additional factors that regulate the *Dstn^corn1^* phenotypes other than SRF, which was previously determined as a major contributor. Further identification of factors that play a role in the formation of *Dstn^corn1^* phenotypes will provide valuable insights into the regulation of actin dynamics, absence of vasculature, controlled epithelial proliferation and inflammatory response in the cornea.
